# Torsion of an Accessory Liver Lobe in a Newborn

**DOI:** 10.1055/s-0043-1778663

**Published:** 2024-01-15

**Authors:** Tobias Krause, Dietmar Cholewa, Benjamin Liniger, Steffen Berger, Milan Milosevic

**Affiliations:** 1 Department of Pediatric Surgery, Inselspital Universitätsspital Bern, Bern, Switzerland

**Keywords:** accessory liver lobe torsion, hepatic lobe, neonatal liver lobe torsion, pedunculated liver lobe

## Abstract

Accessory liver lobes are rare. We present the rare case of torsion of an accessory liver lobe in a neonate.

A 13-day-old newborn presented with failure to thrive and hematemesis without fever. The initial workup with sonography, magnetic resonance imaging, and upper gastrointestinal study was suspicious of a duplication cyst, most likely in the posterior wall of the stomach. Laboratory and radiological findings were not suggesting a choledochal cyst. We performed a laparotomy with resection of the 3.2 × 2.1 × 1.1 cm mass. Intraoperatively, the cystic formation extended from of the liver bed up to the lesser curvature of the stomach. The mass was attached to the left liver lobe with fibrous bands. Histopathology revealed necrotic liver parenchyma with patent viable biliary ducts, indicative of an accessory liver lobe that underwent torsion in the perinatal period. The postoperative course and follow-up (6 months so far) were uneventful. To our knowledge, this is the youngest described patient in the literature with an accessory liver lobe torsion and the second case report concerning this entity in a neonate. It presents an extremely rare differential diagnosis in symptomatic neonates with a cystic mass in the upper abdomen.

## Introduction

Accessory liver lobes (ALLs) are anatomic variations in the liver with heterogeneous clinical features. While they may remain asymptomatic, they can also lead to severe symptoms, particularly when torsion occurs. 1 2 3 4 ALLs are rare, and the majority of reported cases involve infants, adolescents, or adults. 4 5 6 7 We report on an extremely rare case of a symptomatic ALL due to a torsion in a neonate.

## Case Presentation

A 13-day-old newborn was referred from another hospital with failure to thrive due to poor drinking behavior, two episodes of hematemesis on the 7th day of life, and recurrent bilious vomiting since then. There were no congenital conditions found in the past family history. The pregnancy progressed without complications, with routine prenatal sonography checkups revealing no abnormalities. The neonate was born at full term with a gestational age of 40 weeks and 6 days, and a birthweight of 4,100 g. There were no perinatal abnormalities and both stool and urine exhibited normal color.

The physical examination revealed unremarkable results, except for a low weight of 3,790 g, representing a 7.6% weight loss since birth.

Laboratory data indicated normal liver function tests with aspartate aminotransferase, alanine aminotransferase, alkaline phosphatase, gamma glutamyl transpeptidase, Quick and international normalized ratio all within the normal range. No anemia was present. Slight increases in total bilirubin (22 µmol/L [norm < 17 µmol/L]) and direct bilirubin (7 µmol/ L [norm < 5 µmol/L]) were observed. Catecholamines in the urine, as well as tumor markers for alpha-fetoprotein and beta chorionic gonadotropin, fell within the normal range. An increased C-reactive protein of up to 37 mg/L (norm: < 5 mg/L) was noted without clinical signs of infection. Lactate and leukocyte levels remained within the normal range.

**Fig. 1:**
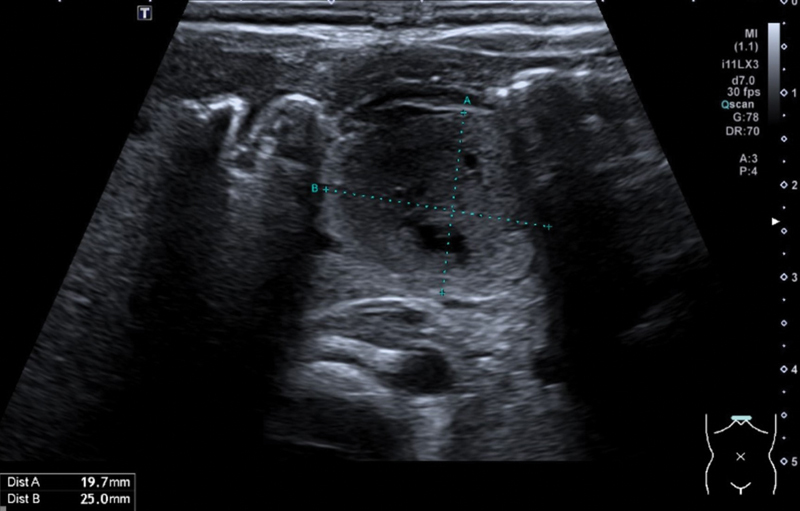
Preoperative ultrasound showing the mass close to the stomach, pancreas, and duodenum.

**Fig. 2:**
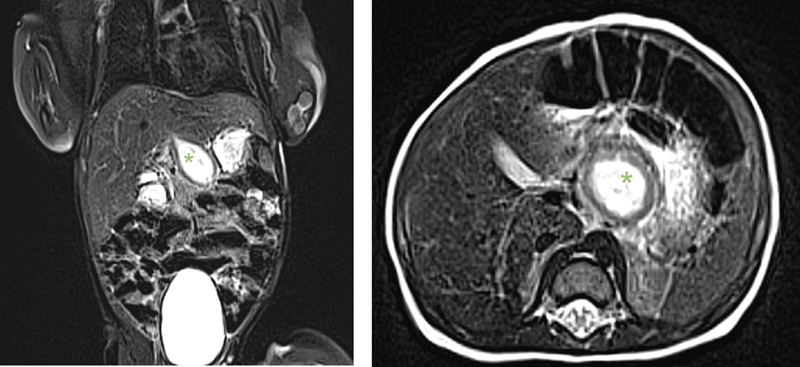
Preoperative T2 image of abdomen with hyperintense cystic formation (*).

Abdominal sonography revealed a 3 × 2.5 × 2 cm cystic mass close to the duodenum, stomach, and pancreas without perfusion. The lesion showed a thick echogenic wall, an intermediate echogenicity with multiple small anechoic cystic inclusions, and an inhomogeneous internal structure. The kidneys, pancreas, spleen, gallbladder, and liver were normal, and we found no signs of dilated biliary ducts ( Fig. 1 ). The subsequent upper gastrointestinal (GI) study showed an impression of the lesser curvature through the cystic mass with no further obstruction. To further delineate the location and origin of the cystic mass, T2-weighted magnetic resonance imaging (MRI) confirmed its proximity to the stomach and liver ( Fig. 2 ).

**Fig. 3:**
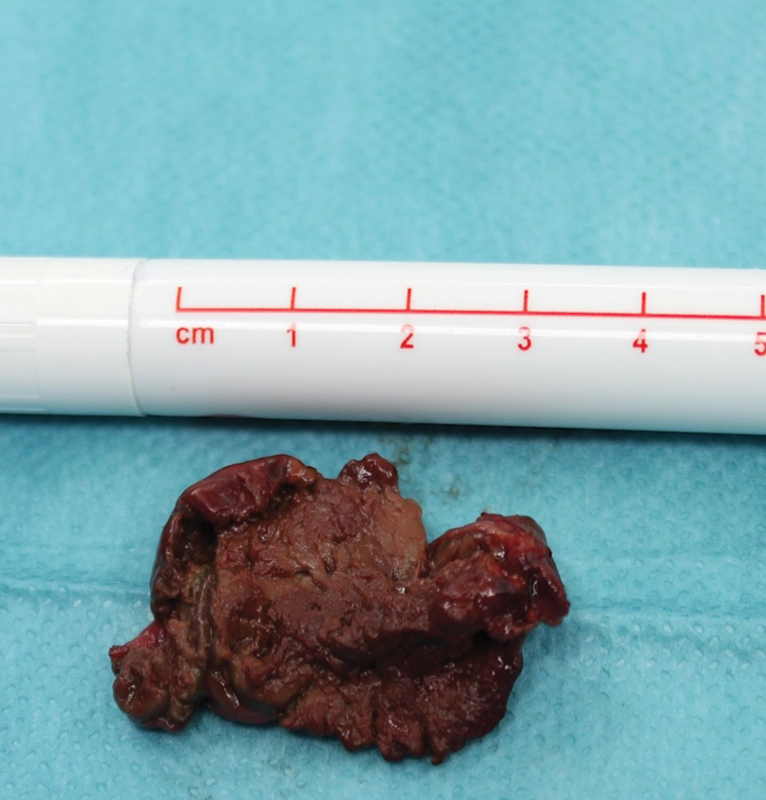
Intraoperative picture of the resected mass.

With a preliminary diagnosis of duplication cyst, we performed a transverse upper abdominal laparotomy. During the procedure, we located the cystic mass posterior to the lesser curvature of the stomach ( Fig. 3 ). Surprisingly, the mass was attached to the liver by a thin fibrotic stalk. Neither the stomach nor the biliary tree was involved and therefore, simple resection of the lesion was feasible.

Histopathology revealed necrotic liver parenchyma with a few vital bile ducts, coated with liver capsule. The histological findings were primarily consistent with a hepatic infarction, so the diagnosis of torqued ALL was made.

The perioperative course proceeded without complications.

The patient was discharged on the sixth postoperative day on full enteral feeds without vomiting and an increase in his bodyweight up to 3,970 g. After a follow-up of 6 months, the neonate was doing well.

## Discussion

The first description of an ALL was published by Morgagni in 1767. 8 However, the most well-known type is the tongue-like elongation of the hepatic segments V and VI named after Riedel. 9 10 However, case reports, particularly in young children, are rare. To date, only one case involving a 23-day old male neonate with an isolated twisted ALL has been published previously. 11

The most common used classification of ALLs based on the publication of Collan et al 12 :

1. ALL attached to the liver by a stalk
2. ALL attached to the liver by a stalk less than 30 g
3. Ectopic liver without connection to the liver
4. Microscopic ectopic liver

Glenisson et al 10 proposed an alternative classification, which categorizes ALLs into (A) sessile lobes (Riedel's lobe), (B) pedunculated lobes (attached to the liver with a stalk), and (C) ectopic lobes. According to these classifications, the specimen in our case falls into the ALL type 1/2 and type B, respectively.

The presence of an ALL may be linked to anterior abdominal wall defects. 3 13 14 15 In the case report and literature review conducted by Corbitt et al, 16 it was found that among 22 children, nine had associated abdominal wall defects, such as omphalocele, bladder exstrophy, and umbilical hernia. Notably, our patient did not manifest any further malformations.

Symptomatic cases require surgical treatment; however, whether asymptomatic and incidentally discovered ALLs should be removed remains unclear. Given the potential risks, including torsion or the development of hepatocellular carcinoma, 17 18 a surgical approach appears to be a reasonable consideration. Ladurner et al even reported a case of a complete hepatic ischemia resulting from the torsion of a large ALL, necessitating liver transplantation. 19

The preferred surgical approach in the literature is heterogeneous, but laparoscopy seems to be feasible and safe for small ALLs. 1 2 11 In retrospect, the pedunculated lobe in our patient probably could have been removed laparoscopically despite the young age of our patient.

ALLs are challenging to diagnose prior to surgery because preoperative imaging and laboratory tests often yield inconsistent results. Like many cases of ALL in the literature, our diagnosis was made intraoperatively and confirmed through histopathology. 20 However, the use of sonography, MRI, and upper GI studies is valuable for excluding differential diagnoses with potentially more severe short- and long-term sequelae, such as cystic biliary atresia, volvulus, and choledochal cyst.

## Conclusion

To the best of our knowledge, this case represents the youngest described patient with torsion of an ALL and is only the second documented case in a neonate. 11 Torsion of ALLs should be considered a differential diagnosis in symptomatic neonates with cystic masses in the upper abdomen. Resection in the neonatal age is feasible and safe.
